# Expression of testicular phosphorylated proteins in types 1 and 2 diabetes mellitus in mice: An experimental study

**DOI:** 10.18502/ijrm.v17i8.4822

**Published:** 2019-09-03

**Authors:** Apichakan Sampannang, Supatcharee Arun, Jaturon Burawat, Wannisa Sukhorum, Sitthichai Iamsaard

**Affiliations:** ^1^Department of Anatomy, Faculty of Medicine, Khon Kaen University, Khon Kaen Thailand.; ^2^School of Medicine, Mae Fah Luang University, Chiang Rai Thailand.; ^3^Research Institute for Human High Performance and Health Promotion (HHP&HP), Khon Kaen University, Khon Kaen Thailand.

**Keywords:** Diabetes mellitus, Testicular phosphorylated protein, Steroidogenic acute regulatory protein, Streptozotocin, Mice

## Abstract

**Background:**

Types 1 and 2 diabetes mellitus (DM) are known to be the cause of sub/infertility. However, the comparisons of potential markers in spermatogenesis and steroidogenesis in DM males have never been elucidated.

**Objective:**

This study aimed to examine the expressions of tyrosine-phosphorylated and steroidogenic acute regulatory (StAR) proteins in testis of DM mice.

**Materials and Methods:**

Fifty-six male C57BL/6 mice were divided into four groups (n░=░14/ each): control of MLD-STZ (multiple low doses of streptozotocin), MLD-STZ, control of HFD-STZ (high-fat diet with STZ), and HFD-STZ. MLD-STZ mice (type 1 DM) were intraperitoneally (i.p.) injected with STZ at 40░mg/kg BW for five days. HFD-STZ mice (type 2 DM) received an HFD for 14 days and i.p.-induced by STZ at 85░mg/kg BW and fed with HFD. At the end of the experiment (days 36 and 72), the expressions of phosphorylated proteins and StAR were examined.

**Results:**

Tyrosine phosphorylated proteins were localized in late spermatids, luminal fluid, and Leydig cells. The intensities of phosphorylated 110, 85, 72, 60, and 55░kDas were lower in the 36 day-DM mice. Although such intensities were present in both groups, only 85░kDa in the MLD-STZ mice was higher in HFD mice at 72 days. StAR expressions in both groups were decreased than that of the controls.

**Conclusion:**

Decreased expressions of StAR and tyrosine-phosphorylated proteins may be directly involved in low testosterone levels and impaired spermatogenesis. These findings support the notion that both DM types play a role in male infertility.

## 1 Introduction

Diabetes mellitus (DM; classified into types 1 and 2) is a metabolic disorder characterized by hyperglycemia. Diabetes mellitus can induce functional and structural damages to various organs (1). In addition, complications from types 1 and 2 DM have detrimental effects on the reproductive system (2, 3). Previous studies in DM men and experimental animals have demonstrated the molecular impairment of reproductive functions (4–6). For example, it has been demonstrated that DM affects the expression of testicular proteins including that of the steroidogenic acute regulatory (StAR) protein (7) and vascular endothelial growth factor (8). However, potential alterations to other markers in DM testis still need to be investigated in order to better understand the mechanisms that influence infertility in male patients with types 1 and 2 DM. Many testicular proteins have been reported to play roles in testosterone production and spermatogenesis. Specifically, the doublesex- and mab-3-related transcription factor 1 (DMRT1) is expressed in the spermatogonia playing roles in male sex determination and differentiation (9). In spermatocytes, DAZL, a well-known RNA-binding protein is involved in testicular differentiation, proliferation, and meiosis (10). Moreover, SPAG17 found in spermatids are involved in the conversion of nucleosomal chromatin and sperm development and maturation (11). It has recently been found that tyrosine-phosphorylated proteins are specifically located in Sertoli cells and late spermatids (4, 12, 13). Previous studies have suggested that phosphorylated proteins may play important roles in spermatogenesis and testosterone production (5, 6, 14–17). Protein phosphorylation is essential for sperm capacitation and acrosome reaction in the early stages of fertilization (18, 19). Interestingly, it has been demonstrated that the patterns of testicular phosphorylated proteins can change under various inductions (i.e., drugs, stressors, or chemotherapeutic reagents), and these changes are associated with increases in endogenous acrosome reactions and sperm abnormalities (5, 6, 14–17). Such protein changes are also related to decreases in StAR protein levels, which is widely used as a marker to assess testosterone production (7, 15, 16). Changes in testicular phosphorylated proteins may also be a cause of DM-related male infertility. As described earlier, there has yet never been a report that compares the adverse effects on male reproductive parameters, especially testicular protein markers, between types 1 and 2 DM.

This study investigated these parameters, including the expression of testicular tyrosine-phosphorylated and StAR proteins, in mice with type 1 DM-induced by multiple low doses of streptozotocin (MLD-STZ) and type 2 DM-induced by a high-fat diet (HFD) with streptozotocin (HFD-STZ).

## 2 Materials and Methods

### 2.1 Animals and diabetes induction

Male C57BL/6 mice (n░=░56) were obtained from the Nomura Siam International Co., Ltd. (Bangkok, Thailand). Animals were accommodated in ventilated cages and had ad-libitum access to a commercial pellet diet and water under a 12-h light/ dark cycle at the Northeast Laboratory Animal Center, Khon Kaen University, Thailand. The mice were divided into four groups: (1) multiple low-dose of streptozotocin (MLD-STZ) control, (2) MLD-STZ, (3) HFD co-treated with streptozotocin (HFD-STZ) control, and (4) HFD-STZ groups (n░=░14 per group). Before the experiment, all mice were starved for 16░hours. In control, mice of the MLD-STZ model were intraperitoneally (i.p.) injected with 0.1░M citrate buffer (pH░=░4.5) for five consecutive days. Whereas, MLD-STZ mice (type 1 DM) were induced with STZ (Sigma-Aldrich, USA) at a dose of 40░mg/kg BW for five consecutive days via i.p. injection (20). The control mice of the HFD-STZ model received a normal diet (10░kcal % fat, D12450░J, Research Diet, Inc., USA) for 14 consecutive days and were then i.p. injected with 0.1░M citrate buffer (pH░=░4.5). In type 2 DM model, HFD-STZ mice received an HFD (60░kcal % fat, D12492, Research Diet, Inc., USA) for 14 consecutive days and were then induced with a single dose of STZ at a dose of 85░mg/kg BW (21) as well as continuously fed an HFD as previously described (22). At days 3, 6, 12, 18, 24, 30, 36, 42, 48, 54, 60, 66, and 72 after STZ induction, the mice of both groups underwent blood glucose measurement via tail prick and using a blood glucose oxidase reaction monitoring system to confirm their diabetic conditions. The animals were considered to be diabetic when their blood glucose levels were greater than 250░mg/dl. All animals were maintained for 36 and 72 days (one and two spermatogenesis cycles) (23).

### 2.2 Immunohistochemistry

After fixation, all testicular tissues were dehydrated with ascending series of alcohols, cleared in xylene, infiltrated, and embedded with melted paraffin using the auto-processor apparatus at, Department of Pathology, Faculty of Medicine, to make the tissue blocks. Then, the paraffin blocks were cut at 5–7░mm thickness (Semi-automatic Rotary Microtome, ERM 3100 HESTION, Australia). The tissue sections were further rehydrated and antigenic retrieved by soaking in citrate buffer (10░mM citric acid, 0.05 % Tween-20, pH 6.0) before heating with microwave at 95░°C. To block the endogenous peroxidase activity, the tissue section was incubated with 30% hydrogen peroxide before blocking of non-specific proteins with 5% bovine serum albumin (BSA; Millipore Co., USA). Then, four sections were incubated with monoclonal phosphotyrosine antibody (clone 4G10, 1:200 (v/v); Millipore, Co., USA) while the negative control group was omitted for the primary antibody. All sections were washed with PBS and then incubated with secondary antibody (horseradish peroxidase-conjugated goat anti-mouse IgG (1:300 (v/v); Invitrogen^TM^, USA). To detect the Ag-Ab complex, the sections were incubated with a kit of the Vector NovaRED peroxidase substrate (Vector Laboratories, USA). Hematoxylin was used as a counterstained dye. The immunoreactive testicular sections was photographed using a Nikon light ECLIPSE E200 light microscope equipped with a DXM1200 digital camera (Nikon, Japan).

### 2.3 Testicular protein preparation and western blot analysis

Total protein concentration of the testicular lysate was measured at an absorbance of 280░nm by using a NanoDrop ND-1000 Spectrophotometer (NanoDrop ND-1000 Spectrophotometer V3.5, USA). The testicular proteins were separated on 10% sodium dodecyl sulfate-polyacrylamide gel electrophoresis (SDS-PAGE) and further transferred onto nitrocellulose membranes. Protein membranes were then incubated with 3% skim-milk blocking solution for 2░hr at room temperature. After washing with 0.05% PBST (PBS, 0.05% (v/v) Tween-20), all membranes were incubated with primary antibody (phosphotyrosine 4G10 antibody, 1:1000 (v/v); Millipore Co., USA), anti-StAR antibody (1:1000 (v/v); Santa Cruz Biotechnology, USA), or anti-*β*-actin antibody (1:1000 (v/v); Santa Cruz Biotechnology, USA; used as an internal control). Then, the washed membranes were incubated with specific secondary antibody (conjugated with HRP) before washing with 0.05% PBST. The expressions of such target proteins were detected by using an enhanced chemiluminescence (ECL) substrate reagent kit (GE Healthcare Life Science, USA) performed in the Image Quant 400 imaging system (GH Healthcare Life Sciences, USA). The intensity of individual protein bands were quantified by using ImageJ program. The epidermal growth factor (EGF stimulated A413 cell lysate; Millipore Co., USA) and StAR lysate (Catalogue no. sc-112333; Santa Cruz Biotechnology Inc., USA) were used as positive controls while bovine serum albumin (BSA; Millipore Co., USA) was used as a negative control.

### 2.4 Ethical consideration

This study was approved by the Animal Ethics Committee of Khon Kaen University, based on the Ethics of Animal Experimentation as determined by the National Research Council of Thailand (ref. No. 0514.1.75/90 with record No. AEKKU-NELAC 71/2559).

### 2.5 Statistical analysis

All data were subjected to the Shapiro-Wilk test (W-test) to evaluate the normal distribution and equality of variances. The one-way ANOVA was used to compare the mean values for normally distributed data using the SPSS Statistics (Statistical Package for the Social Sciences, version 19.0, SPSS Inc., Chicago, Illinois, USA). A p░<░0.05 was considered to indicate a significant difference. All data were expressed as mean░±░standard deviation (SD).

## 3 Results

Immunohistochemical localization of tyrosine phosphorylated proteins in testicular sections from STZ-treated mice.

**Figure 1:**
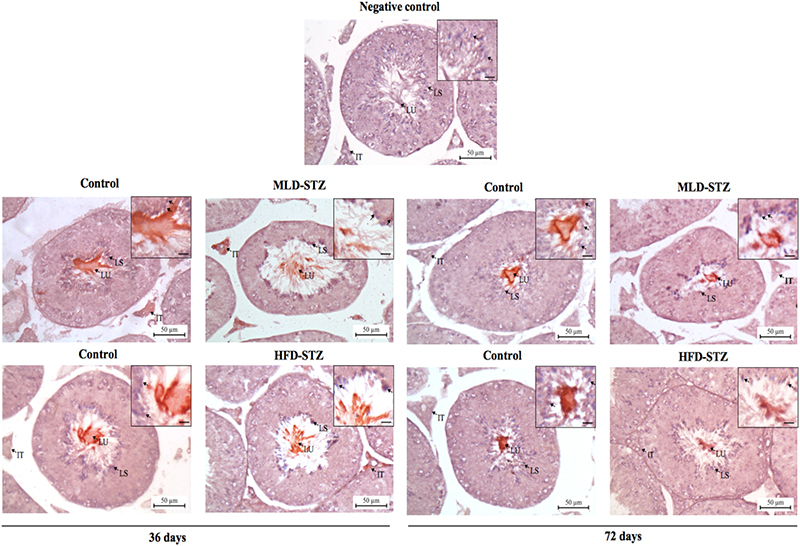
Representative immunohistochemical localization of tyrpsine-phosphorylated proteins in seminiferous epithelium and interstitial tissue compared among control, MLD-STZ, and HFD-STZ groups at experimental days 36 and 72. The dark red areas represent positive immunoreactivity. IT: interstitial tissue; LS: Late spermatids; LU: luminal fluid; Scale bar: 10░*μ*m.

Tyrosine-phosphorylated proteins in the MLD-STZ and HFD-STZ mice were specifically localized within late spermatids, fluid in the seminiferous lumen, and interstitial tissue (cytoplasm of Leydig cells) as compared to that of omitted-primary antibody control (Figure 1). The amounts of positive-phosphorylated proteins in both MLD-STZ and HFD-STZ testes seemed to be lower than those in the control testes at both day 36 and day 72 (Figure 1).

### 3.1 Effect of MLD-STZ and HFD-STZ on the expression of testicular steroidogenic acute regulatory (StAR) protein

**Figure 2:**
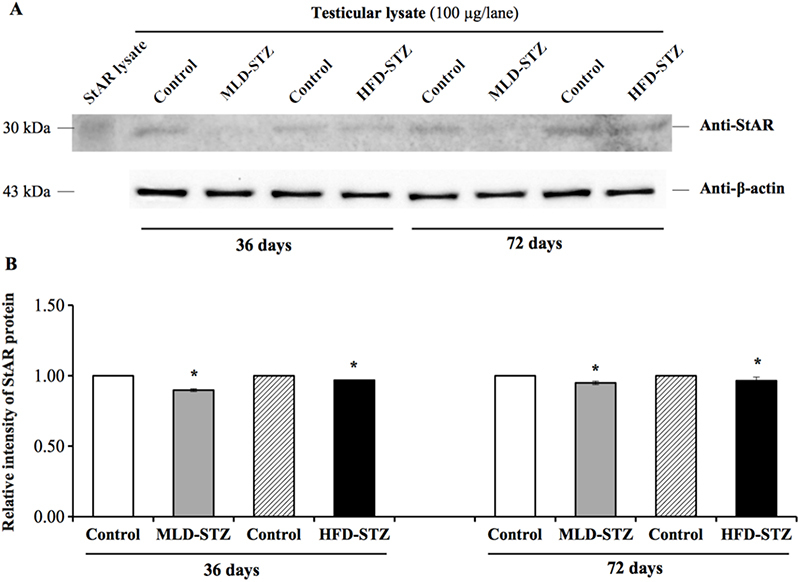
Representative immuno-western blot (A) and intensity (B) of testicular steroidogenic acute regulatory (StAR) protein expression in the control, MLD-STZ, and HFD-STZ groups at experimental days 36 and 72. StAR lysate was used as a positive control. *β*-actin was used as an internal control protein. Data are represented as mean░±░SD. ^*^P *<* 0.05 compared with control.

Using *β*-actin as an internal control protein, we found that the expression of the StAR protein in MLD-STZ and HFD-STZ testis tended to be lower than those in the controls at both 36 and 72 days (Figure 2A). In addition, the relative intensity of StAR expression in both STZ groups was significantly lower than that of the control at both day 36 and day 72 (p░≤░0.001, 0.003, Figure 2B).

### 3.2 Effect of MLD-STZ and HFD-STZ on expression patterns of tyrosine-phosphorylated proteins in the testicular lysate

**Figure 3:**
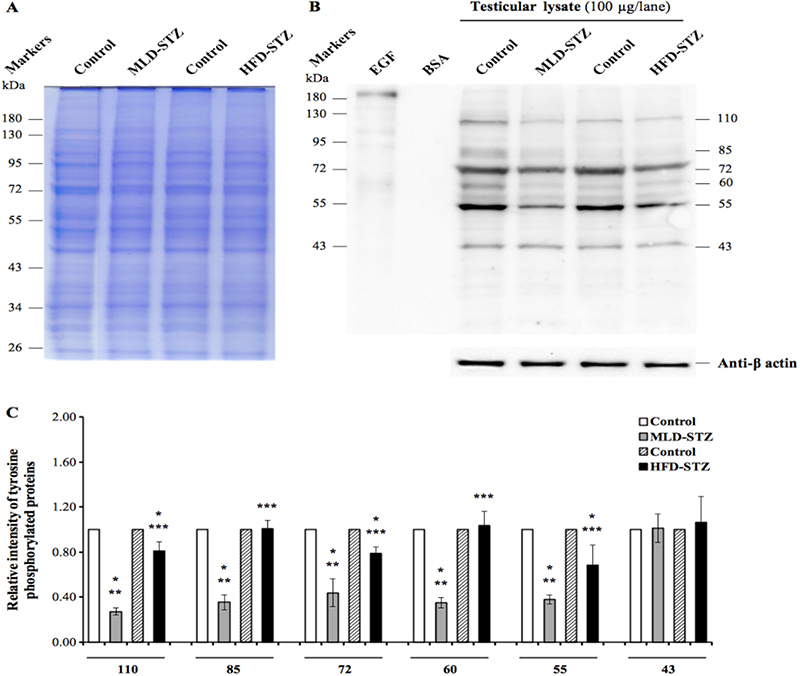
Representative testicular protein profiles revealed by sodium dodecyl sulfate-polyacrylamide gel electrophoresis (SDS-PAGE) at day 36 (A), immuno-western blot of tyrosine-phosphorylated proteins in testicular lysates (B), and relative intensity of testicular-phosphorylated proteins (110, 85, 72, 60, 55, and 43░kDas; (C) compared among the control, MLD-STZ, and HFD-STZ groups. Epidermal growth factor (EGF)-like growth factor was used as a positive control and bovine serum albumin (BSA) as a negative control. *β*-actin was used as an internal control. Data are represented as mean░±░SD. *P < 0.05 compared with control. **P < 0.05 compared with HFD-STZ. ***P < 0.05 compared with MLD-STZ.

**Figure 4:**
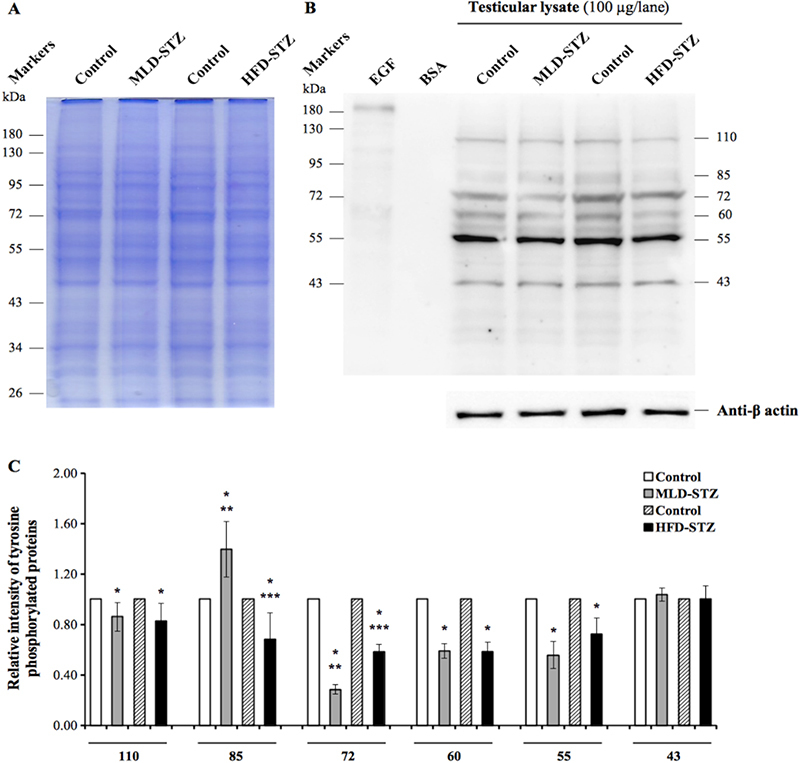
Representative testicular protein profiles revealed by sodium dodecyl sulfate-polyacrylamide gel electrophoresis (SDS-PAGE) at day 72 (A), immuno-western blot of tyrosine-phosphorylated proteins in testicular lysates (B), and relative intensity of testicular-phosphorylated proteins (110, 85, 72, 60, 55, and 43░kDas; (C) compared among the control, MLD-STZ, and HFD-STZ groups. Epidermal growth factor (EGF)-like growth factor was used as a positive control and bovine serum albumin (BSA) as a negative control. *β*-actin was used as an internal control. Data are represented as mean░±░SD. ^*^P *<* 0.05 compared with control. ^**^P *<*0.05 compared with HFD-STZ. ^***^P *<* 0.05 compared with MLD-STZ.

Equal amounts of testicular proteins were confirmed using SDS-PAGE and *β*-actin expression (Figures 3A-B, 4A-B). On days 36 and 72 of the experiment, the result showed six major phosphorylated proteins (110, 85, 72, 60, 55, and 43░kDas) of testicular lysate in both STZ inductions (Figures 3B, 4B). Intensity analysis revealed that five band protein expression (110, 85, 72, 60, and 55░kDas) at days 36 and 72 differed (Figures 3C, 4C). The relative intensity of 110░kDa-phosphorylated protein in the MLD-STZ and HFD-STZ groups was significantly lower at 36 days than that of the control (p░≤░0.001, 0.005, Figures 3C). Although the intensity of 110░kDa protein in the HFD-STZ group was significantly higher than that of MLD group at day 36 (p░≤░0.001, Figure 3C), there was no difference at day 72 (p░=░0.576, Figure 4C). The intensity of 85░kDa in the MLD-STZ group was significantly lower than that of the control at day 36 (p░≤░0.001, Figure 3C) but significantly higher at day 72 (p░=░0.001, Figure 4C). The intensity of 85░kDa-phosphorylated protein in the HFD-STZ group did not differ from that of the control at day 36 (p░=░0.943, Figure 3C) but was significantly lower at day 72 (p░=░0.003, Figure 4C). At day 36, the intensity of 85░kDa-phosphorylated protein in the MLD group was significantly lower than that of the HFD group (p░≤░0.001, Figure 3C). However, it was significantly higher than that of a 85░kDa in MLD-STZ group at day 72 (p░≤░0.001, Figure 4C). The relative intensity of 72░kDa protein in both groups was significantly lower at days 36 and 72 compared to that of the control (p░≤░0.001, Figures 3C, 4C). The intensity of this protein in the MLD-STZ group was significantly lower than that in the HFD-STZ group at both experimental days (p░≤░0.001, Figures 3C, 4C). Additionally, 60░kDa protein intensity at days 36 and 72 was significantly lower in MLD-STZ group than in the control (p░≤░0.001, Figures 3C, 4C). The intensity of a 55░kDa protein in both groups was also significantly lower than that of the control (p░≤░0.001, Figures 3C, 4C). The intensities of 60 and 55░kDa proteins of MLD-STZ group at day 36 were significantly lower than those of the HFD-STZ group (p░≤░0.001, Figure 3C), but at day 72, this difference of 60░kDa protein had vanished (p░=░0.945, Figure 4C). In contrast, 55░kDa protein intensity in MLD-STZ group at day 72 was significantly lower than that of the HFD-STZ group (p░=░0.035, Figure 4C). We also found that there was no difference among all groups in terms of 43░kDa protein intensity (Figures 3C, 4C).

## 4 Discussion

A recent study has explained the testicular histopathology and phosphorylated protein changes in ICR-outbred mice with DM-induced by MLD-STZ, but some reproductive parameters have yet to be elucidated (6). These outbred mice may have lower STZ induction than the inbred strains commonly used as diabetes and obesity models (24, 25). Gurley and co-researchers (2006,^24^) reported the level of STZ sensitivity in several strains of mice to be as follows: DBA/2░>░C57BL/6 > MRL/MP░>░129/ SvEv░>░BALB/c. Therefore, we have used C57BL/6 mice instead of ICR mice in this study. Recently, MLD-STZ injection has become widely used to induce type 1 DM in animal models (6, 20, 24) because it can mimic human DM (20). Additionally, the combination of an HFD and a single low dose of STZ injection can more closely mimic human type 2 DM than that in nicotinamide (NA)-STZ models (2, 21, 22).

Steroidogenic acute regulatory (StAR) protein is commonly used as a marker for the assessment of the testosterone production in the testes (7, 15). Previous studies have demonstrated reductions in serum testosterone, LH, and FSH levels in both type 1 (4, 7) and 2 DM (2, 26). Our study showed that the intensity of StAR protein expression was lower in MLD-STZ mice, similar to that of rats given one high dose (OHD) STZ (7). However, its expression was lower than found in a previous study (7). At day 72, StAR expression in the MLD-STZ group had decreased to a level lower than that reported by Xu et al. (7). Moreover, the lower levels of this protein in HFD-STZ mice in both experiments were consistent with the findings of a previous report in NA-STZ rats (26).

High insulin has been shown to downregulate mRNA of StAR, P450scc, 3*β*- and 17*β*-HSD in HFD mice (27, 28), HFD-STZ (29), and NA-STZ rats (26). This indicates that testicular steroidogenesis is downregulated in animals on an HFD. The decrease of StAR expression in our HFD-STZ model was similar to that previously found in an obesity model (7), in that it resulted in decreases in testosterone levels (2, 4, 7, 26).

Tyrosine-phosphorylated proteins are specifically located within testicular tissues (12) and are assumed to play a role in spermatogenesis (5, 6, 14–17). However, a report showed that protein phosphorylation was practically absent in the interstitial cells of OHD-STZ DM rats (4). Our study demonstrated for the first time that there were decreases in the positive immunoreactivity of such proteins in the late spermatids, luminal fluid, and interstitial tissue in both groups of DM mice. It also is the first to demonstrate five different protein bands (110, 85, 72, 60, and 55░kDas) in MLD- and HFD-STZ C57BL/6 mice. The expression of 66 and 50░kDa proteins was similar to that in OHD-STZ rats (4). Previously, we reported that testicular 70░kDa was higher in OHD-STZ mice than in control (5). Herein, the novelty of this study is that type 1 DM animals in 36 days have significant decrease of testicular-phosphorylated proteins compared to those of type 2 DM mice. In 72 days, only 72 testicular protein was significantly increased in type 2 DM when compared with that of type 1 DM. The changes of testicular phosphorylation were found more in type 1 DM animals. Previous studies have shown that protein phosphorylation is essential for sperm capacitation and acrosome reaction (18, 19). Impaired protein phosphorylation leads to male infertility (18, 19). Although the phosphorylation found in late spermatids, seminiferous tubule fluid, and interstitial tissue in this study has yet to be explained, we suspect that the different localization in many other studies might be due to different specificity of antibodies or animal strains (12). The fluid secreted by the Sertoli cells contains nutritional and hormonal microenvironment substances (30). This may include tyrosine-phosphorylated protein since it is localized in Sertoli cells (12). Tyrosine phosphorylation imbalance in interstitial tissue can also decrease testosterone production (4).

## 5 Conclusion

This study found that type 1 and type 2 DM changed the expressions of tyrosine-phosphorylated and StAR proteins in mouse testes. Testicular phosphorylated proteins were expressed only in Sertoli cells and interstitial tissue in the DM models. The changes in 110, 85, 72, 60, and 55░kDas-phosphorylated and StAR proteins found in DM testes might be associated with the suppression of spermatogenesis, resulting in male sub/infertility.

## Conflict of Interest

The authors of this article have no conflicts of interest.
